# *Hspb1* inhibits microglial ferroptosis and pro-inflammatory activation to alleviate cerebral ischemia/reperfusion injury in mice

**DOI:** 10.4103/NRR.NRR-D-24-01532

**Published:** 2025-08-13

**Authors:** Weilong Hua, Hongye Xu, Rundong Chen, Yiyong Zeng, Lei Zhang, Yongxin Zhang, Xiaoxi Zhang, Yongwei Zhang, Hongjian Zhang, Jianmin Liu, Pengfei Yang

**Affiliations:** 1 Neurovascular Center, Changhai Hospital, Naval Medical University, Shanghai, China; 2 Department of Neurology, No. 904 Hospital of the PLA Joint Logistics Support Force, Wuxi, Jiangsu Province, China; 3 School of Health Science and Engineering, University of Shanghai for Science and Technology, Shanghai, China; 4 Department of Neurosurgery, the First Affiliated Hospital of Ningbo University, Ningbo, Zhejiang Province, China; 5 Oriental Pan-Vascular Devices Innovation College, University of Shanghai for Science and Technology, Shanghai, China; 6 Department of Neurosurgery, Shidong Hospital Affiliated to University of Shanghai for Science and Technology, Shanghai, China

**Keywords:** cerebral ischemia/reperfusion, heat shock protein beta-1, inflammatory, microglial ferroptosis, nuclear factor-κB/glutathione peroxidase 4 signaling axis

## Abstract

Heat shock protein beta-1 may be involved in regulating ferroptosis in cells. The expression of heat shock protein beta-1 is upregulated after stroke; however, the underlying mechanism of action of heat shock protein beta-1 in cerebral ischemia/reperfusion injury remains unclear. Here, using both *in vivo* and *in vitro* models of ischemic injury—middle cerebral artery occlusion/reperfusion in C57BL/6J mice and oxygen-glucose deprivation/reoxygenation in BV-2 microglial cells—we observed that heat shock protein beta-1 overexpression significantly reduced infarct volume, mitigated neuronal loss, and improved neurological outcomes. Mechanistically, heat shock protein beta-1 attenuated lipid peroxidation, intracellular iron accumulation, and reactive oxygen species generation in microglia; this was accompanied by enhanced glutathione peroxidase 4 expression and suppressed nuclear factor-κB pathway activation. Notably, the pharmacological activation of nuclear factor-κB with phorbol 12-myristate 13-acetate reversed the protective effects of heat shock protein beta-1, confirming the functional relevance of this pathway. Together, our findings indicate that heat shock protein beta-1 exerts neuroprotective effects against cerebral ischemia/reperfusion injury by suppressing microglial ferroptosis and pro-inflammatory activation via modulation of the nuclear factor-κB/glutathione peroxidase 4 signaling axis. These findings establish heat shock protein beta-1 as a critical regulator of the nuclear factor-κB/glutathione peroxidase 4 axis in microglia, thereby offering a dual-targeted strategy to inhibit ferroptosis and inflammation in ischemic stroke. Importantly, our study highlights heat shock protein beta-1 as a promising therapeutic candidate for preserving neurological function following cerebral ischemic injury.

## Graphical abstract

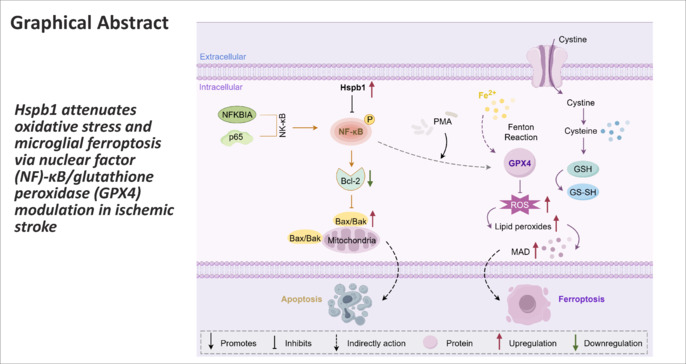

## Introduction

Stroke continues to be a leading cause of mortality worldwide, imposing a substantial global burden (Feigin et al., 2022). Cerebral ischemic injury is a prevalent pathological condition that is observed in cases of ischemic stroke; its underlying mechanisms encompass an inflammatory response, oxidative stress, excitatory amino acid toxicity, cellular apoptosis, and ferroptosis (Zhang et al., 2024). Ferroptosis refers to cell death triggered by increased lipid peroxidation that is induced by iron accumulation (Hu et al., 2024). This process plays a pivotal biological role in the initiation and progression of cerebral ischemia/reperfusion (I/R) injury. The precise connections and mechanisms between ferroptosis and necroinflammation in cerebral ischemia-related disorders have not yet been fully explored (Proneth and Conrad, 2019). It is therefore imperative to investigate the importance and associated regulatory targets of ferroptosis in the pathological progression of ischemic stroke. This exploration may yield novel approaches and concepts for clinical treatments and related ailments.

Currently, intravenous thrombolysis and mechanical thrombectomy have become effective methods for restoring blood flow to brain tissue within the specified time window to rescue the ischemic penumbra tissue (PEN) (Hankey, 2017; Ermine et al., 2021; Jin et al., 2022). However, when reoxygenation occurs after ischemia, it leads to I/R injury and causes additional tissue damage in PEN because of the production of reactive oxygen species (ROS) and subsequent inflammation (Wu and Prentice, 2021). Ultimately, this results in neuronal cell dysfunction and death (Orellana-Urzúa et al., 2023). Microglia-mediated inflammation plays a crucial role in I/R-induced cerebral injury (Wu et al., 2018; Zheng et al., 2024; Chen et al., 2025). Microglia are the resident immune cells of the central nervous system, and have an important role in maintaining homeostasis and responding to injury (Zhang et al., 2025). Traditionally, microglial activation has been classified into “resting” and “activated” states, or further categorized into M1 and M2 phenotypes. However, recent studies challenge these simplistic classifications, highlighting the plasticity of microglial states that depend on the specific injury context and surrounding microenvironment (Paolicelli et al., 2022). This understanding is critical for accurately interpreting microglial functions in various pathological conditions. Understanding the molecular pathways that regulate microglial polarization is thus crucial for developing therapeutic strategies to mitigate cerebral I/R injury.

A key molecular node that may link inflammation and ferroptosis is the nuclear factor (NF)-κB/glutathione peroxidase (GPX4) signaling axis. NF-κB is a transcription factor that is broadly implicated in inflammatory responses; its activation is an early hallmark of ischemic injury (Yan et al., 2021). By contrast, GPX4 is a vital antioxidant enzyme that suppresses ferroptosis by reducing lipid hydroperoxides. Emerging evidence indicates that NF-κB may negatively regulate GPX4 expression, thus facilitating ferroptotic vulnerability under pathological conditions (Shi et al., 2023). However, it remains poorly understood how this axis may be modulated in microglia during I/R injury.

Neuronal cells respond to stress events by activating their own protective mechanisms, such as by increasing the production of heat shock proteins (HSPs), suppressing inflammation, and preventing cell death (Stetler et al., 2012). HSPs have cytoprotective properties and exhibit different expression patterns in various tissues (Kirbach and Golenhofen, 2011). In the brain, all HSPs have been observed to undergo phosphorylation during ischemia. Among these proteins, HSP beta-1 (HSPB1) appears to play a crucial role in protecting neurons from damage caused by I/R injury, and may contribute to neuroprotection (Bartelt-Kirbach et al., 2017). Ischemic encephalopathy is characterized by reduced blood flow to the brain, leading to neuronal damage and dysfunction. Previous studies indicate that HSPB1 can act as a negative regulator of ferroptotic cancer cell death (Sun et al., 2015; Liang et al., 2023), suggesting its potential involvement in regulating ferroptosis. However, some studies have reported that HSPB1 is upregulated in response to stroke. These apparently contradictory results suggest that the mechanisms underlying the role of HSPB1 in stroke remain unclear, and indicate that further research is needed to clarify the specific function of HSPB1 in ischemic stroke.

The middle cerebral artery occlusion with reperfusion (MCAO/R) model in rodents is widely recognized as a reliable and easily reproducible representation of human cerebral ischemia (DeVries et al., 2001). This model typically induces substantial damage in the cortex and caudate putamen, leading to impaired sensorimotor behavior, which has been extensively investigated in studies of ischemic stroke models (Nagel et al., 2004).

Despite these findings, research has not yet addressed whether HSPB1 modulates ferroptosis and neuroinflammation through the NF-κB/GPX4 signaling pathway in the context of cerebral I/R injury. Given the central role of microglia in post-ischemic neuroinflammation and ferroptosis, we hypothesized that HSPB1 may exert neuroprotection by attenuating microglial ferroptosis and pro-inflammatory activation via inhibition of the NF-κB/GPX4 axis. To test this hypothesis, we used a combination of *in vivo* (MCAO/R mouse model) and *in vitro* (oxygen-glucose deprivation/reoxygenation [OGD/R] BV-2 microglial cell model) approaches. The present study aimed to elucidate the previously uncharacterized molecular mechanisms by which HSPB1 modulates glial ferroptotic-inflammatory crosstalk, thus offering novel insights into potential therapeutic targets for ischemic stroke.

## Methods

### Ethics statement

All animal procedures were approved by the Animal Experimentation Ethics Committee of Ningbo University (No. AEWC-NBU20230325) and conducted in strict accordance with the National Institutes of Health Guide for the Care and Use of Laboratory Animals (8^th^ ed., National Research Council, 2011). All experiments were designed and reported according to the Animal Research: Reporting of *In Vivo* Experiments (ARRIVE) guidelines (Percie du Sert et al., 2020).

### Establishment of the middle cerebral artery occlusion with reperfusion model

C57BL/6J mice raised under specific pathogen-free conditions were obtained from Charles River Laboratories (Zhejiang, China) under license No. SCXK (Zhe) 2019-0001. The mice were 8 weeks old and weighed 26–28 g. Only male mice were used to avoid estrogen-related neuroprotection that may confound stroke outcomes. Measures were taken to minimize the number of animals used and their discomfort. All mice were housed under standard conditions with a controlled temperature (24 ± 2°C), relative humidity (55% ± 5%), and a 12-hour light/dark cycle. They had unrestricted access to standard chow and water. Prior to establishing the model for mimicking ischemic stroke *in vivo* using MCAO/R as previously described (Wu et al., 2022), the mice underwent a fasting period of 12 hours. Mice were then anesthetized with 1.5%–2.0% isoflurane delivered via inhalation using a precision vaporizer (RWD Life Science, Shenzhen, China) and placed in a supine position. Body temperature was maintained at 36°C using a heating pad. A midline incision was performed on the neck to expose the right common carotid artery, external carotid artery, and internal carotid artery. After partially ligating the common and internal carotid arteries, a vascular incision was made at the distal end of the external carotid artery. Through this incision, a single-strand nylon thread (diameter: 0.22 mm) was inserted along the external carotid artery and into the internal carotid artery to induce occlusion of the MCA. After 60 minutes, reperfusion was initiated by withdrawing the nylon thread. Mice were provided *ad libitum* access to food in their cages. The success of the I/R model was determined using a laser speckle flow imager (SIM BFI-ZOOM) as shown in **Additional Figure 1**. To ensure the reliability of the results, only animals that were successfully reperfused were included in the final analysis. Mice in the sham group underwent identical surgical procedures without occlusion of the MCA origin.

**Figure 1:**
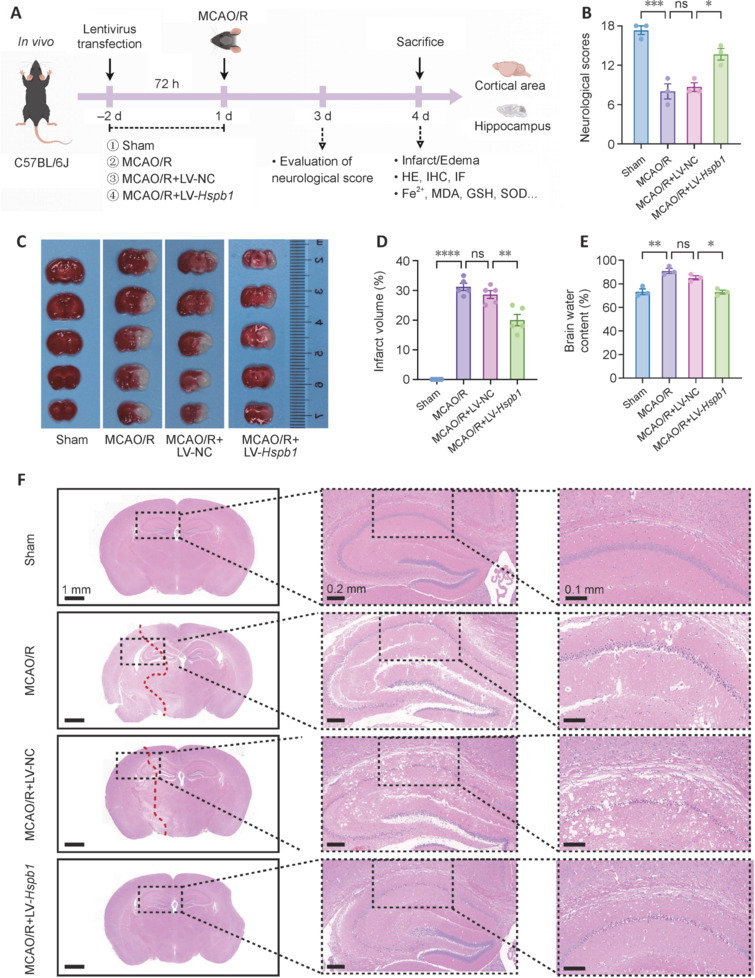
Protective effect of *Hspb1* against ischemic reperfusion injury in mice subjected to MCAO/R. (A) *In vivo* experimental outline. Sham, no treatment; MCAO/R group, middle cerebral artery occlusion/reperfusion without treatment; MCAO/R + LV-NC group, lentiviral vector negative control; and MCAO/R + LV-*Hspb1* group, MCAO with overexpression of *Hspb1* using lentivirus. (B) Neurological behavior was assessed using a score ranging from 0–18. *n* = 3 per group. (C) Representative sections stained with 2,3,5-triphenyltetrazolium chloride (TTC). *n* = 5 per group. White indicates the infarct region. (D) Quantification of infarct volume using TTC staining. The bar graph represents the percentage of infarct area relative to the total brain volume. *n* = 5 per group. (E) Determination of brain water content. *n* = 3 per group. (F) Representative images of hematoxylin and eosin staining. *n* = 3 per group. The red dotted line distinguishes between the infarcted and normal areas. Data are shown as mean ± SEM. *P < 0.05, **P < 0.01, ***P < 0.001, ****P < 0.0001 (one-way analysis of variance followed by Tukey’s *post hoc* test). Hspb1: Heat shock protein beta-1; I/R: ischemia/reperfusion; MCAO/R: middle cerebral artery occlusion/reperfusion; ns: not significant; OGD/R: oxygen-glucose deprivation/reoxygenation.

### Animal grouping and administration

The mice were randomly divided into four groups: the Sham, MCAO/R, MCAO/R + lentiviral vector negative control (LV-NC), and MCAO/R + lentiviral vector overexpressing *Hspb1* (LV-*Hspb1*) groups (*n* = 8 mice/group). To administer the treatment, LV-NC or LV-*Hspb1* (5 × 10^8^ TU/mL lentivirus/injection in a volume of 10 μL) was administered via intracerebroventricular injection 72 hours prior to inducing MCAO at the following stereotaxic coordinates: anteroposterior –0.22 mm, mediolateral ±1.0 mm, and dorsoventral –2.5 mm relative to bregma. Coordinates were determined using the Mouse Brain Atlas by Paxinos and Franklin (2019). The size of the infarct caused by MCAO was assessed using 2,3,5-triphenyltetrazolium chloride (TTC) staining (Sigma, St. Louis, MO, USA). Modified Neurological Severity Scores (mNSS) were used to evaluate post-stroke behavioral impairments.

### Identification and dissection of the ischemic penumbra

The ischemic penumbra was identified as the tissue regions surrounding the core infarct area that were affected by ischemic injury but not yet completely infarcted. The tissue was first subjected to TTC (Sigma) staining, in which viable tissue is stained red and infarcted areas remain pale. The penumbra, characterized by an area of reduced blood flow and viable but compromised tissue, was carefully dissected from the surrounding cortex and used for further protein expression analysis. Tissue samples from both the infarcted area and the penumbra were collected separately to evaluate the protein expression of apoptosis-related and inflammatory genes.

### Assessment of neurological deficits

Mice underwent mNSS assessments on days 1 and 3 post-MCAO, following previously described methodology (Sugawara et al., 2008). The mNSS evaluation encompasses tests for motor function (muscle condition and abnormal movement), sensory perception (visual, tactile, and proprioceptive abilities), reflexes, and balance. A score is assigned when a test cannot be performed or if a reflex response is absent. A higher score indicates more severe neurological impairment (normal score: 0; maximum score: 18).

### Measurement of the infarct area

Seventy-two hours after cerebral I/R, mice were euthanized with a terminal overdose of isoflurane (5% in oxygen, via inhalation) until a loss of reflexes was confirmed; this was followed by cervical dislocation to ensure death. Next, excised brains were promptly submerged in phosphate-buffered saline (PBS) at 4°C for 15 minutes. Subsequently, specimens were sectioned coronally at 2 mm thickness using a tissue slicer (ZS-QGZ, ZSbio, Beijing, China). Sections were then incubated in 2% TTC (Sigma) solution at 37°C for 15 minutes. Digital photographs of the stained tissue slices were acquired for analysis, and the areas of infarction were identified and demarcated. The extent of infarction in each brain section was quantified using Image-Pro Plus 7.0 software (Media Cybernetics, Rockville, MD, USA). To do this, we deducted the area of undamaged tissue in the left hemisphere from the corresponding region in the right hemisphere, thereby controlling for the effects of cerebral edema. The measurements of infarct area were conducted blindly and are presented as a percentage of the total area of the ipsilateral hemisphere. The formula used for the calculation was as follows:


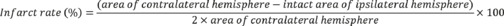


### Brain edema assay

A separate group of mice was used to assess brain edema using the wet-dry technique. The formula for the calculation was as follows:


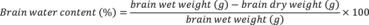


### Hematoxylin and eosin staining

Mouse brain tissue was rapidly harvested, sectioned into 5-mm coronal slices, washed with saline solution, and fixed in paraformaldehyde overnight. The samples then underwent a gradual dehydration process through increasing concentrations of alcohol (70%, 80%, 90%, and 95% for 2 hours, 1.5 hours, 1 hour, and 1 hour, respectively) followed by two 30-minute periods in each of anhydrous alcohol and xylene. Next, the specimens were infiltrated with paraffin in three 30-minute intervals. The solidified tissue was embedded using a heated paraffin embedding system (Leica, Wetzlar, Germany) and sectioned into 5-μm slices using a Semi-Automated Rotary Microtome (Leica). The sections then underwent a staining protocol involving two 10-minute xylene baths, sequential alcohol dehydrations in decreasing concentrations for 3 minutes each, and washing with running water. Hematoxylin and eosin staining (Beyotime, Shanghai, China) followed; this included a series of quick dips in acid alcohol and a bluing reagent, coupled with brief water rinses. Finally, the stained slices were again dehydrated through a series of alcohols, cleared in xylene, and mounted with neutral resin. The prepared hippocampal sections were then examined under an Inverted Research Microscope (Nikon, Tokyo, Japan) to observe any pathological changes.

### Immunohistochemistry

After performing antigen retrieval using citrate buffer (Beyotime), brain sections were treated with 3% H_2_O_2_ for 15 minutes. Subsequently, the sections were incubated with QuickBlock™ Blocking Buffer (Beyotime, P0260) at room temperature (25 ± 3°C) for 30 minutes. The sections then underwent overnight incubation at 4°C with the primary antibody rabbit anti-caspase-3 (CASP3; 1:100, Abcam, Cambridge, UK, Cat# ab32351, RRID: AB_725946). Next, the slides were washed twice with PBS before being incubated with biotinylated goat anti-rabbit immunoglobulin (Ig)G (H+L) secondary antibody (1:500 dilution, Vector Laboratories, Newark, CA, USA, Cat# BA-8000, RRID: AB_2336140) for 30 minutes at room temperature. This was followed by incubation with horseradish peroxidase (HRP)-conjugated streptavidin (Vector Laboratories) for 30 minutes at room temperature. Hematoxylin counterstaining was then performed, and immunoreactivity was visualized using 3,3′-diaminobenzidine substrate (Zsbio). An optical microscopy (Zeiss, Oberkochen, Germany) was used to capture images, which were then analyzed using ImageJ (National Institutes of Health, Bethesda, MD, USA).

### Cell culture and *in vitro* oxygen-glucose deprivation/reoxygenation model

The BV-2 mouse microglial cell line (accession number ATL03001, RRID: CVCL_0182, ICLC repository) was used. The BV-2 cells were suspended and cultured in Dulbecco’s Modified Eagle Medium (DMEM; Thermo Fisher Scientific, Waltham, MA, USA, Cat# C11995500BT) supplemented with 10% fetal bovine serum (FBS; Thermo Fisher Scientific, Cat# 10099141) and 1% antibiotic-antimycotic (Thermo Fisher Scientific, Cat# 15240062). They were maintained in a humidified incubator at 37°C with 5% CO_2_. To create an OGD/R cellular model, the BV-2 cells were transferred to glucose-free and FBS-free DMEM (Sigma). Subsequently, they were placed in a hypoxic incubator at 37°C with 5% CO_2_ and 95% N2 for 6 hours. This was followed by two 24-hour incubations in complete medium under normal conditions (5% CO_2_, 95% O_2_, 37°C) for reperfusion. Cells in the control group were incubated in complete medium under normal conditions throughout the entire experiment. The BV-2 cells were randomly assigned to the following experimental groups to assess the effects of *Hspb1* overexpression and NF-κB activation under OGD/R conditions: (1) control group, cultured under normoxic conditions without any treatment; (2) LV-NC group, transduced with LV-NC under normoxia; (3) LV-NC+PMA group, transduced with LV-NC and treated with 80 nM phorbol 12-myristate 13-acetate (PMA, an NF-κB agonist; Sigma); (4) OGD/R group, subjected to oxygen-glucose deprivation followed by reoxygenation to mimic I/R injury; (5) OGD/R + PMA group, treated with 80 nM PMA after OGD/R; (6) OGD/R + LV-*Hspb1* group, transduced with LV-*Hspb1* prior to OGD/R; and (7) OGD/R + LV-*Hspb1* + PMA group, transduced with LV-*Hspb1*, followed by OGD/R and treatment with 80 nM PMA. The LV-*Hspb1* and LV-NC were purchased from Genechem Co., Ltd., (Shanghai, China). In the OGD/R + PMA group and OGD/R + LV-*Hspb1* + PMA group, BV-2 cells were exposed to 80 nM PMA for 24 hours. In the latter group, BV-2 cells underwent pretreatment through transfection with lentivirus at a multiplicity of infection of 10. Subsequently, successfully transfected cells were collected for quantitative reverse transcription-polymerase chain reaction (qRT-PCR) and western blot analysis. Both LV-*Hspb1*-transfected BV-2 cells and LV-NC-transfected BV-2 cells (serving as negative controls) were preserved by freezing for further experiments.

### Lentiviral vector construction and transduction

Both LV-*Hspb1* and LV-NC were designed and synthesized by Genechem Co., Ltd. The lentiviral sequences are shown in **Additional Table 1** and **Additional Figure 2**. For the *in vitro* experiments, BV-2 cells were seeded into six-well plates at a density of 1 × 10^5^ cells/well in 2 mL of complete medium, and were incubated at 37°C in 5% CO_2_ until they reached 30% confluence. Lentivirus was added at a multiplicity of infection of 10. The cells were incubated for 12 hours, after which the medium was replaced with fresh complete medium. Transduction efficiency was assessed using a fluorescence microscopy (Zeiss) 72 hours after infection. Successfully transduced cells were selected with puromycin (5 µg/mL) for 7 days.

**Additional Table 1:** Sequences of Hspb1 lentivirus

Name	Forward primer	Reverse primer
Hspb1	GAGGTATCCTGACCCTGAAGTA	CACACGCAGCTCATTGTAGA

Hspb1: Heat shock protein beta-1.

**Figure 2:**
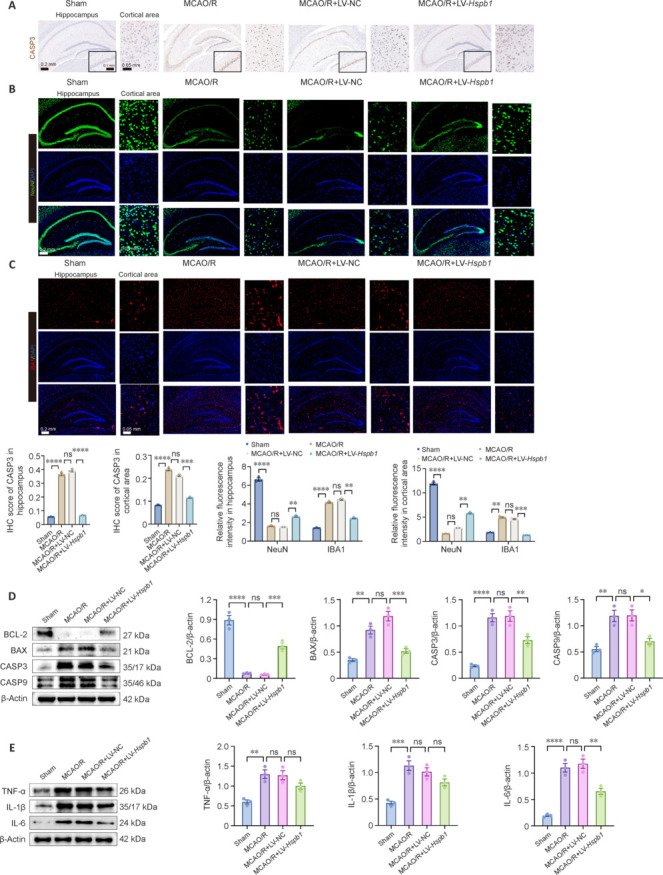
Impact of *Hspb1* on the expression of neuronal proteins and apoptosis- and inflammation-associated proteins in cerebral tissue. (A) Representative images of caspase (CASP)3 immunohistochemistry staining in the hippocampus and cortical area. *n* = 3 per group. (B) Immunoreactivities of neuronal nuclei (NeuN) and (C) ionized calcium binding adaptor molecule 1 (IBA1) in the hippocampus and cortical area, determined using immunofluorescence. *n* = 3 per group. (D) Representative protein bands of apoptosis-related genes were analyzed quantitatively, including apoptosis regulator BCL-2, apoptosis regulator BAX, CASP3, and CASP9. (E) Representative protein bands of genes related to inflammation and the quantitative analysis of tumor necrosis factor (TNF)-α, interleukin (IL)-1β, and IL-6. *n* = 3 per group. Data are shown as mean ± SEM. *P < 0.05, **P < 0.01, ***P < 0.001, ****P < 0.0001 (one-way analysis of variance followed by Tukey’s *post hoc* test). CASP: Caspase; DAPI: 4′,6-diamidino-2-phenylindole; Hspb1: heat shock protein beta-1; IBA-1: ionized calcium-binding adapter molecule 1; IL: interleukin; LV-Hspb1: lentiviral vectors overexpressing Hspb1; LV-NC: lentiviral vectors carrying a negative control; MCAO/R: middle cerebral artery occlusion/reperfusion; NeuN: neuronal nuclei; ns: not significant; TNF-α: tumor necrosis factor-α.

For the *in vivo* studies, lentiviral vectors were intracerebroventricularly injected into the right hemisphere of mice (5 × 10^8^ TU/mL in 10 μL) using stereotactic surgery. Injections were performed 72 hours before inducing ischemic injury to ensure the stable overexpression of *Hspb1*. Lentivirus administration was followed by the monitoring of neurological and histological outcomes.

### Detection of lipid-derived reactive oxygen species

Lipid ROS levels were measured using a BODIPY-581/591-C11 assay kit (Abclonal, Wuhan, China). BV-2 cells were seeded into six-well plates at a density of 5 × 10^4^ cells/well and incubated for 24 hours. Next, we added BODIPY-581/591-C11 at a concentration of 2 µM to the cells, which were then incubated in darkness at 37°C for 30 minutes. To remove any excess BODIPY-581/591-C11, the cells underwent two washes with PBS. The fluorescence intensity was then analyzed using an inverted fluorescence microscope (Zeiss) with ImageJ. The fluorescence imaging used conventional filters: Texas Red (wavelengths of 581–591 nm) and fluorescein isothiocyanate (FITC; wavelengths of 488–510 nm). Simultaneously, we acquired data for oxidized BODIPY at an excitation maximum wavelength of 488 nm and emission maximum wavelength of 510 nm.

### Detection of intracellular reactive oxygen species

The BV-2 cells were cultured in 12-well plates with a seeding density of 5 × 10^5^ cells/well. Initially, the cells underwent various treatments. Subsequently, cells were incubated in a solution containing 6-carboxy-2’,7’-dichlorodihydrofluorescein diacetate (Thermo Fisher Scientific, Cat# C400) at a concentration of 10 μM for 30 minutes at 37°C without exposure to light. Cells were then rinsed twice with PBS and harvested using trypsinization. Flow cytometry was used to measure fluorescence levels using an emission wavelength of 495 nm and an absorption wavelength of 525 nm.

### Immunofluorescence

Brain sections underwent antigen retrieval using citrate buffer (Beyotime), which was typically performed by heating at 95°C for 10–20 minutes. The sections were then treated with QuickBlock^TM^ Blocking Buffer (Beyotime, Cat# P0260) at room temperature for 30 minutes. Subsequently, sections were incubated overnight at 4°C with primary antibodies against CASP3 (rabbit monoclonal, 1:100, Abcam, Cat# ab32351, RRID: AB_725946), HSPB1 (rabbit monoclonal, 1:500, Abcam, Cat# ab109376, RRID: AB_10865046), GPX4 (mouse monoclonal, 1:400, Santa Cruz Biotechnology, Dallas, TX, USA, Cat# sc-166570, RRID: AB_2112427), NF-κB p65 (rabbit monoclonal, 1:250, Abcam, Cat# ab32536), neuronal nuclei (NeuN; mouse monoclonal, 1:200 Cat# MAB377, EMD Millipore, Beijing, China), cluster of differentiation CD86 (rat monoclonal, 1:500, Abcam, Cat# ab119857, RRID: AB_10902800), CD206 (rabbit monoclonal, 1:400, Cell Signaling Technology, Cat# 24595, RRID: AB_2892682), and ionized calcium-binding adapter molecule 1 (IBA-1; rabbit monoclonal, 1:100, Cat# ab178846, Abcam). Corresponding secondary antibodies (Alexa Fluor 488/594 goat anti-rabbit IgG [H+L]; 1:100, A-11008/A-11012, Invitrogen or Alexa Fluor 488 goat anti-mouse IgG [H+L]; 1:100, A-11001, Invitrogen or Invitrogen or Alexa Fluor 488 goat anti-rat IgG [H+L]; 1:100, A-11006, Invitrogen) were then applied. After two 20-minute washes in PBS-Tween and a counterstaining step with 4′,6-diamidino-2-phenylindole (1:2000, ab104139, Abcam) for 5 minutes, images were captured using a Zeiss HB050 inverted microscope system. Fluorescence intensity was measured using ImageJ.

### Western blot analysis

A protein extraction kit (Beyotime) was used to extract total protein from mouse brain tissue and BV-2 cells. Extracted protein concentrations were determined using a bicinchoninic acid assay kit (Beyotime). A suitable quantity of prepared protein sample was then added to a gel for electrophoresis separation and subsequent transfer onto a polyvinylidene fluoride membrane (0000151028, EMD Millipore). Subsequently, the membrane was incubated at 23°C in blocking buffer (5% skim milk in 20 mM Tris-HCl [pH 7.5], 137 mM NaCl, and 0.2% Tween-20) for 1 hour. The membrane was then incubated overnight at 4°C with primary antibodies specific for anti-apoptosis regulator BAX (rabbit monoclonal, 1:1000, Cell Signaling Technology, Cat# 41162, RRID: AB_2924730), anti-apoptosis regulator BCL-2 (mouse monoclonal, 1:1000, Santa Cruz Biotechnology, Cat# sc-492, RRID: AB_2064290), anti-CASP3 (rabbit monoclonal, 1:5000, Abcam, Cat# ab32351), anti-CASP9 (rabbit monoclonal, 1:1000, Abcam, Cat# ab32539, RRID: AB_725960), anti-tumor necrosis factor alpha (TNF-α; rabbit monoclonal, 1:1000, Abcam, Cat# ab183218, RRID: AB_2889388), anti-interleukin (IL)-1β (rabbit polyclonal, 1:1000, Cell Signaling Technology, Cat# 2022, RRID: AB_2124464), anti-IL-6 (rabbit monoclonal, 1:1000, Abcam, Cat# ab233706, RRID: AB_2889391), anti-HSPB1 (rabbit monoclonal, 1:1000, Abcam, Cat# ab109376), anti-cystine/glutamate transporter SLC7A11 (rabbit monoclonal, 1:1000, Abcam, Cat# ab307601, RRID: AB_3094570), anti-GPX4 (rabbit monoclonal, 1:1000, Abcam, Cat# ab125066), anti-NF-κB p65 (rabbit monoclonal, 1:1000, Abcam, Cat# ab32536, RRID: AB_776751), anti-phosphorylated NF-κB p65 (p-p65) (rabbit polyclonal, 1:1000, Cell Signaling Technology, Cat#3031, RRID: AB_330559), anti-IκBα (rabbit monoclonal, 1:1000, Cell Signaling Technology, Cat# 9242), anti-phospho-IκBα (rabbit monoclonal, 1:1000, Cell Signaling Technology, Cat# 2859), and anti-β-actin (mouse monoclonal, 1:1000; Servicebio, Wuhan, China, Cat# GB12001). The membranes then underwent three rounds of washing using a preprepared Tris-buffered saline-Tween 20 solution, followed by incubation with HRP-conjugated secondary antibody for 1 hour at room temperature. The secondary antibodies were as follows: goat anti-rabbit IgG HRP-linked antibody (1:5000, Cell Signaling Technology, Cat# 7074, RRID: AB_2099233) and horse anti-mouse IgG HRP-linked antibody (1:5000, Cell Signaling Technology, Cat# 7076, RRID: AB_330924). Protein bands were then imaged using a Bio-Rad Imaging System. The optical density of each target protein band was normalized against the optical density of β-actin, and was subsequently analyzed using ImageJ to determine the relative expression levels of each protein.

### Evaluation of markers for oxidative stress

After centrifugation at 10,000 × *g* for 10 minutes, the levels of superoxide dismutase (SOD; NS-E10253), glutathione (GSH; BG-HUM11118), and malondialdehyde (MDA; NB-E10376) in the microglial cell supernatant were assessed using enzyme-linked immunosorbent assay kits (Novatein Biosciences, Woburn, MA, USA) following the manufacturer’s instructions. Optical density at 450 nm was measured using a microplate reader (Pulang, Nanjing, China).

### Determination of iron levels

After microglial cell supernatants were centrifuged at 10,000 × *g* for 10 minutes, we used an Iron Assay Kit (MIK4893; MesGenBiotech, Shanghai, China) to analyze iron ion contents. The concentrations of iron ions in the cells were measured using a microplate reader (Pulang) at a wavelength of 520 nm. Subsequently, we used cell numbers to normalize the intracellular iron ion levels.

### Assay for cytoplasmic ferrous iron

The presence of ferrous iron (Fe^2+^) in the microglial cytoplasm was detected using a fluorescent probe (FerroOrange; F374, Dojindo, Kumamoto, Japan). The cells were exposed to 1 μM of FerroOrange at 37°C for 30 minutes. Subsequently, cells were gently washed three times with serum-free F12 medium (Thermo Fisher Scientific) containing essential nutrients, amino acids, vitamins, and inorganic salts. Fluorescence images were acquired using a fluorescence microscope equipped with appropriate filter sets for excitation at 542 nm and emission at 580 nm.

### Assay of reactive oxygen species in brain tissue

The level of ROS in brain tissue was detected using the ROS detection kit (DHE fluorescence method) (Servicebio, Cat# G1746). 2μL of DHE probe was mixed thoroughly with 1 mL of PBS to prepare DHE staining working solution. The brain slices were exposed to the working solution for 30 minutes at 37°C. Images were captured using a Zeiss HB050 Inverted microscope system, set the excitation wavelength to 520 nm and the emission wavelength to 605 nm.

### Flow cytometry analysis

To prepare for flow cytometry analysis, BV-2 cells were cultured to a density of 1 × 10^5^ cells/well. Cells were then harvested by trypsinization, as follows: after removing the BV-2 cell culture medium (Modified Eagle Medium [PM150410] + 10% FBS [164210-50] + 1% penicillin/streptomycin [PB180120]), cells were gently rinsed with PBS to remove residual serum, followed by the addition of 0.25% trypsin-ethylenediaminetetraacetic acid solution. The cells were incubated at 37°C for approximately 2–3 minutes until detachment was visually confirmed. Next, the detached cells were collected by adding cold PBS to neutralize trypsin, and the cell suspension was centrifuged at 300 × *g* for 5 minutes at 4°C to acquire cell pellets. The supernatant was discarded, and each cell pellet was gently resuspended in 1 mL of cold PBS, followed by another centrifugation step to ensure the removal of any remaining trypsin.

The cells were then resuspended in 100 μL of Annexin V binding buffer (1×; containing Ca^2+^ ions, required for Annexin V binding). Next, cells were gently mixed with 5 μL of Annexin V-FITC (Tianjin Sungene Biotech Co., Tianjin, China), followed by incubation for 15 minutes at room temperature in the dark to prevent photobleaching. After Annexin V labeling, 5 μL of propidium iodide (PI; Tianjin Sungene Biotech Co.) was added to the suspension and incubated for an additional 5 minutes at room temperature in the dark to allow for PI uptake.

To prepare for flow cytometry analysis, the labeled cells were diluted with 400 μL of binding buffer, gently mixed, and placed on ice to prevent any further changes in staining profiles. The prepared cell suspension was then analyzed using a flow cytometer (Sysmex Partec GmbH, Goerlitz, Germany) equipped with excitation lasers suitable for FITC (488 nm) and PI (561 nm) detection. For each sample, at least 10,000 events were collected to ensure statistical significance. Data acquisition was performed using the designated software (FlowJo, FlowJo LLC, Ashland, OR, USA), and cells were gated to distinguish between live (Annexin V^–^/PI⁻), early apoptotic (Annexin V^+^/PI^–^), late apoptotic (Annexin V^+^/PI^+^), and necrotic (Annexin V^–^/PI^+^) populations based on fluorescence intensity.

### Quantitative reverse transcription-polymerase chain reaction

Total RNA was extracted using TRIzol Reagent (Thermo Fisher Scientific) according to the manufacturer’s instructions. Reverse transcription was performed using an Evo M-MLV RT Kit (AG11711, Accurate Biology, Changsha, China), and qRT-PCR was conducted using a SYBR Green Premix qPCR Kit (AG11718, Accurate Biology) on a QuantStudio 5 Real-Time PCR System (Applied Biosystems, Thermo Fisher Scientific). Primer sequences are provided in **Additional Table 2**. Relative gene expression levels were calculated using the 2^–ΔΔCt^ method, with Gapdh serving as the internal reference gene.

**Additional Table 2:** Primer sequences for quantitative reverse transcription-polymerase chain reaction

Gene	Primer sequence (5’-3’)	
	Forward	Reverse
*Hspb1*	CTCACAGTGAAGACCAAGGAAG	GAGAGATGTAGCCATGTTCGTC
*β-actin*	GAGGTATCCTGACCCTGAAGTA	CACACGCAGCTCATTGTAGA

Hspb1: Heat shock protein beta-1.

### Statistical analysis

Statistical analyses were performed using GraphPad Prism (version 10, GraphPad Software, Boston, MA, USA). Data are presented as the mean ± standard error of the mean. For multiple group comparisons, one-way analysis of variance followed by Tukey’s *post hoc* test was used. A *P*-value < 0.05 was considered significant.

## Results

### *Hspb1* overexpression mitigates brain injury, improves neurological outcomes, and alleviates cerebral pathological changes in mice subjected to middle cerebral artery occlusion with reperfusion

The *in vivo* experimental setup is depicted in **Figure 1A**. To confirm the efficiency of *Hspb1* overexpression in brain tissues, qRT-PCR was performed on RNA extracted from the ischemic penumbra. There was a significant upregulation of *Hspb1* mRNA expression in the MCAO/R + LV-*Hspb1* group compared with the findings in the MCAO/R + LV-NC group (*P* < 0.001), indicating the successful overexpression of *Hspb1* in the target region (**Additional Figures 3** and **4**). Primer sequences used for qRT-PCR are provided in **Additional Table 2**. The sham group did not exhibit any significant behavioral deficits (**Additional Figure 1**). Compared with the sham group, mice that underwent MCAO/R showed noticeable neurological dysfunction, as indicated by lower neurological scores (*P* < 0.001; **Figure 1B**). However, the group treated with LV-*Hspb1* showed less deterioration of neurological behaviors compared with the MCAO/R + LV-NC group (*P* < 0.001; **Figure 1B**). The infarct volume, evaluated using TTC staining at 72 hours post-MCAO/R, was smaller in mice treated with LV-*Hspb1* than in mice who received LV-NC (*P* < 0.001; **Figure 1C** and **D**). These findings highlight the potential therapeutic benefits of LV-*Hspb1* for mitigating neuronal damage caused by ischemic stroke. In addition, the group treated with LV-*Hspb1* showed significantly reduced brain edema compared with the MCAO/R + LV-NC group. This finding is supported by **Figure 1E**, which shows that brain edema was visibly reduced in the LV-*Hspb1*-treated group (*P* < 0.05). Furthermore, the MCAO/R group exhibited significant differences in the hematoxylin and eosin staining of hippocampal tissue compared with the sham group (**Figure 1F**). These differences included condensed nuclei and cytoplasm, increased nuclear basophilia, altered neuronal size and shape, and disrupted cell arrangement. Notably, a marked improvement in neuronal morphology was observed in the MCAO-R + LV-*Hspb1* group compared with the findings in the MCAO/R + LV-NC group. These findings suggest that treatment with LV-*Hspb1* may have a neuroprotective effect against damage induced by cerebral I/R injury.

**Figure 3:**
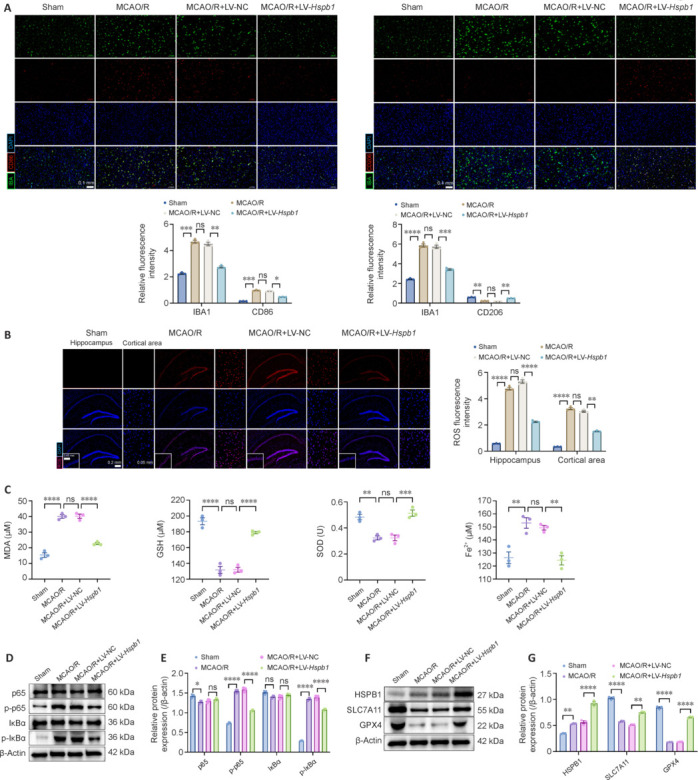
Effects of *Hspb1* on microglia polarization, oxidative stress, and ferroptosis in middle cerebral artery occlusion with reperfusion (MCAO/R) mice. (A) Immunofluorescence of ionized calcium binding adaptor molecule 1 (IBA1) and cluster of differentiation (CD)86/CD206 co-staining in mouse brain tissue. (B) Immunofluorescence of reactive oxygen species (ROS) staining in mouse brain tissue. (C) The levels of malondialdehyde (MDA), glutathione (GSH), superoxide dismutase (SOD), and Fe^2+^ in brain tissue homogenate. *n* = 3 per group. (D, E) Representative protein bands and quantitative analysis of nuclear factor (NF)-κB p65, phosphorylated (p)-p65, IκBα, and p-IκBα. *n* = 3 per group. (F, G) Representative protein bands and quantitative analysis of heat shock protein beta-1 (HSPB1), cystine/glutamate transporter SLC7A11, and glutathione peroxidase (GPX4). *n* = 3 per group. Data are shown as mean ± SEM. *P < 0.05, **P < 0.01, ***P < 0.001, ****P < 0.0001 (one-way analysis of variance followed by Tukey’s *post hoc* test). DAPI: 4′,6-Diamidino-2-phenylindole; GSH: glutathione; Hspb1: heat shock protein beta-1; IBA-1: ionized calcium-binding adapter molecule 1; LV-Hspb1: lentiviral vectors overexpressing Hspb1; LV-NC: lentiviral vectors carrying a negative control; MCAO/R: middle cerebral artery occlusion/reperfusion; MDA: malondialdehyde; NF-κB: nuclear factor kappa-B; ns: not significant; p-: phosphorylated; ROS: reactive oxygen species; SOD: superoxide dismutase.

### *Hspb1* enhances neurological protein expression while reducing apoptosis and the levels of pro-inflammatory cytokines

Following MCAO/R exposure, a significant increase in CASP3 immunoreactivity was observed in both the hippocampus and cortical area (**Figure 2A**). However, LV-*Hspb1* treatment notably ameliorated this effect, particularly in the hippocampus. This finding implies that LV-*Hspb1* may have a protective role against neuronal cell death induced by cerebral ischemia. In addition, NeuN (also known as RNA binding protein fox-1 homolog 3 or RBFOX3) has been extensively studied as a marker for neuronal research within the nervous system. NeuN expression is crucial for identifying and studying neurons in various regions of the brain (Mathern et al., 2007). In the sham group, consistent and abundant NeuN expression was observed throughout specific regions of the hippocampus and cortical area (**Figure 2B**). However, after exposure to MCAO/R injury, there was a noticeable decrease and disruption in NeuN immunoreactivity. This decrease in NeuN immunoreactivity following MCAO/R suggests the existence of compromised neuronal integrity or neurodegeneration. Interestingly, when *Hspb1* was overexpressed (using LV-*Hspb1*), there was a slight increase in NeuN immunoreactivity within the hippocampus and a significant elevation of NeuN immunoreactivity within the cortical area among animals subjected to MCAO/R. This finding implies that *Hspb1* overexpression may have a more pronounced effect on preserving neuronal integrity specifically in this cortical region.

In response to inflammatory signals and various pathological conditions, microglia upregulate IBA1 (also known as allograft inflammatory factor 1 or AIF1). This makes IBA1 a reliable marker for the activation state of microglia (Gheorghe et al., 2023). In the cortical area, LV-*Hspb1* treatment downregulated IBA1 immunoreactivity in the MCAO/R group compared with the findings in the sham group (**Figure 2C**). This finding suggests that LV-*Hspb1* may exert therapeutic benefits by modulating microglial activation and reducing neuroinflammation. To further elucidate the effects of *Hspb1* on cerebral apoptosis and inflammation, western blot analysis was performed to evaluate the expression of the apoptosis-related proteins BCL-2, BAX, CASP3, and CASP9, as well as the inflammation-related proteins TNF-α, IL-1β, and IL-6, in the ischemic penumbra. The protein expression levels of BAX, CASP3, and CASP9 were higher in the MCAO/R group than in the sham group (**Figure 2D**). However, compared with the LV-NC group, the group treated with LV-*Hspb1* showed an effectively reversed upregulation of BAX (*P* < 0.001), CASP3 (*P* < 0.01), and CASP9 (*P* < 0.05) as well as a downregulation of BCL-2 (*P* < 0.001). Additionally, the group exposed to MCAO/R showed a significant increase in the protein levels of TNF-α, IL-1β, and IL-6 compared with the sham group, and *Hspb1* overexpression only partially alleviated this effect on the protein expression of IL-6 (*P* < 0.01; **Figure 2E**). These findings suggest that *Hspb1* overexpression exerts anti-apoptotic effects by downregulating apoptosis-related BAX, CASP3, and CASP9 while upregulating the anti-apoptosis-related BCL2. Furthermore, they indicate that *Hspb1* overexpression may have anti-inflammatory effects by suppressing pro-inflammatory cytokines such as IL-6. Collectively, these results suggest that *Hspb1* may have therapeutic potential for mitigating cerebral tissue damage caused by apoptosis and inflammation associated with ischemic stroke (or other similar neurological conditions).

### *Hspb1* overexpression alleviates the pro-inflammatory activation of microglia, reduces cerebral oxidative stress, and mitigates ferroptosis in the middle cerebral artery occlusion with reperfusion mouse model

We next investigated whether *Hspb1* influences microglial polarization. MCAO/R treatment led to an increased co-expression of IBA1 and CD86 (**Figure 3A** and **B**); this reflects the pro-inflammatory activation of microglia, which is typically associated with inflammatory cytokine release and the exacerbation of neuroinflammation. After LV-*Hspb1* treatment, CD86 expression was reduced, whereas the co-expression of CD206 and IBA1 was increased. CD206 is commonly linked with anti-inflammatory microglia, which contribute to tissue repair and neuroprotection. These changes suggest that LV-*Hspb1* treatment promotes a functional shift in microglia toward a state that supports tissue repair and inflammation resolution. To further investigate the effects of *Hspb1* on oxidative stress in MCAO/R mice, we conducted a comprehensive analysis of various oxidative stress-related indices. First, we observed a notable reduction in relative ROS fluorescence (*P* < 0.001) in the LV-*Hspb1* group compared with that in the LV-NC group (**Figure 3C**). This decrease indicates that *Hspb1* expression leads to a decrease in ROS production, suggesting a potential protective effect against oxidative damage. Second, we noted lower MDA content in the brains of mice treated with LV-*Hspb1* than in those treated with LV-NC (*P* < 0.0001; **Figure 3C**). MDA is an end-product of lipid peroxidation and its reduced levels indicate diminished oxidative damage caused by free radicals. These findings provide additional evidence for the beneficial role of *Hspb1* in mitigating oxidative stress-induced injury. In addition to reduced ROS and MDA levels, we also observed higher levels of GSH (*P* < 0.0001) and SOD (*P* = 0.001) enzymes in brain tissue from the MCAO/R + LV-*Hspb1* group compared with levels in that from the MCAO/R + LV-NC group (*P* < 0.0001; **Figure 3C**). The increased levels of GSH and SOD suggest that antioxidant defense mechanisms are enhanced by *Hspb1* overexpression. Overall, these findings indicate that *Hspb1* exerts inhibitory effects on oxidative stress following MCAO/R injury in mice.

Next, Fe^2+^ concentrations in brain tissue homogenates were measured. LV-*Hspb1* treatment significantly reduced Fe^2+^ levels in the MCAO/R group compared with LV-NC treatment (*P* < 0.01; **Figure 3C**), indicating its potential as a therapeutic intervention for iron overload associated with cerebral I/R injury.

We then investigated the effects of LV-*Hspb1* on key signaling pathways involved in inflammation and cell death. The protein levels of p-p65 and p-NF-κB inhibitor α were both increased after MCAO/R exposure (**Figure 3D** and **E**), suggesting activation of the NF-κB pathway. However, the increase in p-p65 (*P* < 0.0001) and p-NF-κB inhibitor α (*P* < 0.0001) protein expression was reversed by LV-*Hspb1* treatment, indicating that it can inhibit NF-κB signaling and potentially attenuate neuroinflammation. In addition to modulating inflammation, LV-*Hspb1* also exhibited an effect on ferroptosis-related proteins. MCAO/R decreased the protein expression levels of GPX4 and SLC7A11, which are two important markers associated with ferroptosis regulation (**Figure 3F** and **G**). However, *Hspb1* overexpression reversed these MCAO-R-induced changes in the protein expression of GPX4 (*P* < 0.0001) and SLC7A11 (*P* < 0.0001). Together, our findings highlight the anti-oxidative stress and anti-ferroptosis effects of *Hspb1* for mitigating neuronal damage caused by cerebral I/R injury.

### *Hspb1* mitigates oxidative stress, ferroptosis, and the pro-inflammatory polarization of microglia *in vitro*

Microglia are the resident immune cells of the central nervous system; they play a critical role in neuroinflammation, oxidative stress, and ferroptosis. During the pathological process of cerebral I/R injury, microglia are among the earliest responders to damage and are central to both the progression and resolution of inflammation (Lv et al., 2011; Liu et al., 2022). Although previous studies have primarily focused on neuronal HSPB1 expression and its neuroprotective effects, recent research indicates that the sensitivity of microglia to ischemic brain injury also makes them a key cellular model for investigation (Gaire et al., 2018; Zhu et al., 2021). To further validate our rationale for selecting microglia, we conducted dual-label immunofluorescence co-localization experiments with HSPB1 and IBA1. HSPB1 was not only prominently expressed in neuronal cells in ischemic brain tissue but was also clearly localized in microglia (**Additional Figure 5**). This finding further supports the scientific basis for choosing microglia as the focus of our research.

**Figure 4:**
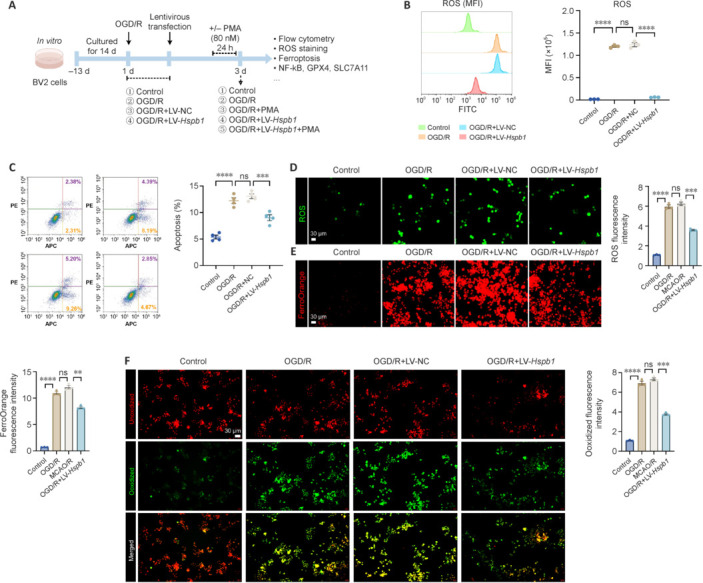
*Hspb1* confers protection against ferroptosis and apoptosis *in vitro* in BV-2 cells. (A) *In vitro* experimental timeline. (B) Reactive oxygen species (ROS) detection using flow cytometry, and the quantitative analysis of mean fluorescence intensity (MFI) of ROS. *n* = 3 per group. (C) Representative figures and quantitative analysis of apoptosis, which was assessed using annexin-V/propidium iodide (PI) staining combined with flow cytometry in BV-2 cells. *n* = 4 per group. (D) Representative fluorescence images of the intracellular ROS activity of BV-2 cells. *n* = 3 per group. (E) Representative images of the fluorescent probe FerroOrange, demonstrating intracellular Fe^2+^ levels, in BV-2 cells. *n* = 3 per group. (F) C11-BODIPY 581/591 probe-stained images in BV-2 cells. *n* = 3 per group. Data are shown as mean ± SEM. ***P < 0.001, ****P < 0.0001 (one-way analysis of variance followed by Tukey’s *post hoc* test). APC: Allophycocyanin; FITC: fluorescein isothiocyanate; GPX4: glutathione peroxidase 4; Hspb1: heat shock protein beta-1; LV-Hspb1: lentiviral vectors overexpressing Hspb1; LV-NC: lentiviral vectors carrying a negative control; NF-κB: nuclear factor kappa B; ns: not significant; OGD/R: oxygen-glucose deprivation/reoxygenation; PE: phycoerythrin; PMA: phorbol 12-myristate 13-acetate (NF-κB agonist); ROS: reactive oxygen species.

**Figure 5:**
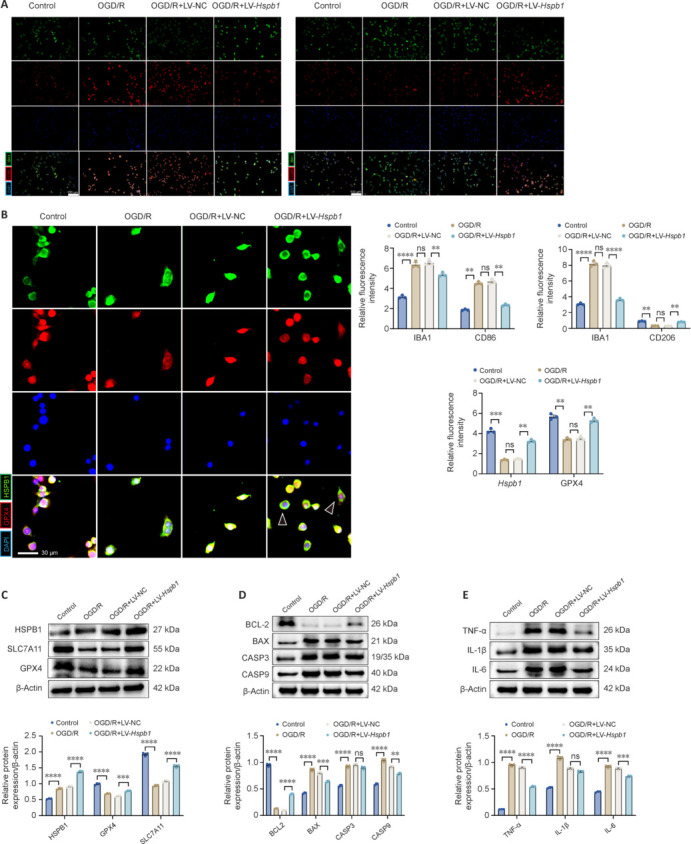
*Hspb1* -induced modulation of the expression of proteins involved in microglia polarization, ferroptosis, and inflammation. (A) Immunofluorescence of ionized calcium binding adaptor molecule 1 (IBA1) and cluster of differentiation (CD)86 or CD206 co-staining in BV-2 cells. *n* = 3 per group. (B) Immunofluorescence of heat shock protein beta-1 (HSPB1) and glutathione peroxidase (GPX4) in BV-2 cells. *n* = 3 per group. Arrowheads identify cells with high co-expression of GPX4 and HSPB1. (C) Western blot measurement and quantitative analysis of HSPB1, cystine/glutamate transporter SLC7A11, and GPX4 protein levels in BV-2 cells. *n* = 3 per group. (D) Western blot analysis was used to measure the protein levels of the apoptosis-related proteins BCL-2, BAX, caspase (CASP)3, and CASP9 in BV-2 cells. *n* = 3 per group. (E) Western blot was used to measure the protein levels of the inflammatory proteins tumor necrosis factor (TNF)-α, interleukin (IL)-1β, and IL-6 in BV-2 cells. *n* = 3 per group. Data are shown as mean ± SEM. **P < 0.01, ***P < 0.001, ****P < 0.0001 (one-way analysis of variance followed by Tukey’s *post hoc* test). DAPI: 4′,6-Diamidino-2-phenylindole; GPX4: glutathione peroxidase 4; IBA-1: ionized calcium-binding adapter molecule 1; IL: interleukin; LV-Hspb1: lentiviral vectors overexpressing Hspb1; LV-NC: lentiviral vectors carrying a negative control; ns: not significant; OGD/R: oxygen-glucose deprivation/reoxygenation; SCL7A: solute carrier family 7; TNF-α: tumor necrosis factor-α.

Our *in vivo* experiments provided evidence to suggest that the synergistic effects of *Hspb1* are associated with its ability to counteract oxidative stress and ferroptosis. To further validate these findings, we conducted *in vitro* experiments to investigate the effects of *Hspb1* overexpression on ROS production and apoptosis in microglial BV-2 cells subjected to OGD/R. A schematic diagram of the *in vitro* model is depicted in **Figure 4A**. Flow cytometry analysis revealed significantly higher (*P* < 0.0001) levels of ROS in the OGD/R group than in the control group (**Figure 4B**). However, when *Hspb1* was overexpressed in the OGD/R + LV-*Hspb1* groups, there was significantly less ROS production compared with that in the OGD/R + LV-NC group (*P* < 0.0001). This finding suggests that *Hspb1* plays a crucial role in reducing OGD/R-induced oxidative stress. Furthermore, there was enhanced resistance to cell death in BV-2 cells overexpressing *Hspb1* compared with that in those cells expressing non-targeting control vectors (OGD/R + LV-NC group, *P* < 0.001; **Figure 4C**). This result indicates that *Hspb1* upregulation confers protection against apoptotic pathways that are triggered by OGD/R insult. To corroborate these findings, we also measured changes in ROS using fluorescence intensity measurements (**Figure 4D**). Consistent with the flow cytometry data, fluorescence intensity was significantly higher (*P* < 0.0001) in the OGD/R group than in the control group. Moreover, BV-2 cells overexpressing *Hspb1* exhibited reduced fluorescence intensity levels, indicating a decrease in ROS accumulation. We also observed a significant increase in intracellular Fe^2+^ levels following OGD/R exposure (**Figure 4E**), indicating enhanced ferrous ion accumulation. However, this effect was notably attenuated upon LV-*Hspb1* transfection. To further explore OGD/R-induced oxidative stress and its modulation by LV-*Hspb1* treatment, we used the C11-BODIPY 581/591 fluorescence probe to detect lipid ROS (**Figure 4F**). Interestingly, we observed that BV-2 cells treated with OGD/R exhibited higher green fluorescence intensity than that of control cells, suggesting elevated lipid ROS production. This finding suggests that OGD/R exposure induces oxidative damage that specifically targets lipids within cells. As expected, LV-*Hspb1* treatment effectively mitigated the OGD/R-induced lipid ROS generation, as evidenced by reduced green fluorescence intensity. Collectively, these findings indicate that *Hspb1* overexpression protects against ferroptosis-induced damage by suppressing lipid peroxidation and subsequent ROS production.

To further investigate the effects of HSPB1 on the pro-inflammatory polarization of microglia and ferroptosis, we conducted double immunofluorescence staining in BV-2 cells. The cells exhibited pro-inflammatory activation after OGD/R treatment, whereas LV-*Hspb1* treatment promoted anti-inflammatory polarization (**Figure 5A**). Following OGD/R, there was a significant upregulation (*P* < 0.0001) of HSPB1 in the LV-*Hspb1* group compared with the findings in the LV-NC group (**Figure 5B**), and this HSPB1 upregulation was accompanied by the elevated expression of GPX4, a marker of ferroptosis. Conversely, both HSPB1 and GPX4 showed low expression levels in the LV-NC group. These findings suggest that under normal conditions or during OGD/R stimulation, there is a downregulation of these proteins, which may contribute to an increased susceptibility to ferroptosis. Consistent with the aforementioned *in vitro* experiments, OGD/R stimulation resulted in reduced protein levels of not only HSPB1 but also SLC7A11 and GPX4 (**Figure 5C**). When *Hspb1* was overexpressed through LV-*Hspb1* transduction into BV-2 cells subjected to OGD/R stimulation, protein levels were restored for all three markers: HSPB1 (*P* < 0.0001), SLC7A11 (*P* < 0.0001), and GPX4 (*P* = 0.001; **Figure 5C**). These findings indicate that *Hspb1* overexpression can rescue the downregulated expression levels induced by OGD/R, and may enhance cellular defense mechanisms against ferroptosis. Moreover, we observed that *Hspb1* upregulation partially mitigated the suppressive effects of OGD/R stimulation on BAX (*P* < 0.001) protein expression, while also enhancing the expression levels of BCL-2 (*P* < 0.0001), CASP3 (*P* < 0.01), and CASP9 (*P* < 0.01) proteins (**Figure 5D**). In addition to the observed effects on apoptosis-related proteins, OGD/R stimulation also affected pro-inflammatory protein expression in microglial cells. Specifically, OGD/R stimulation led to a significant upregulation of TNF, IL-1β, and IL-6 (**Figure 5E**). However, when *Hspb1* was upregulated in addition to OGD/R stimulation, the upregulation of pro-inflammatory proteins such as TNF (*P* < 0.0001) and IL-6 (*P* < 0.001) was attenuated. Overall, these findings highlight the therapeutic potential of modulating *Hspb1* expression in microglial cells for protecting against ferroptosis-induced cell death and apoptosis, and also possibly dampening excess inflammation.

### *Hspb1* upregulation may attenuate ferroptosis in microglial cells through inhibition of the nuclear factor-κB signaling pathway

**Figure 6:**
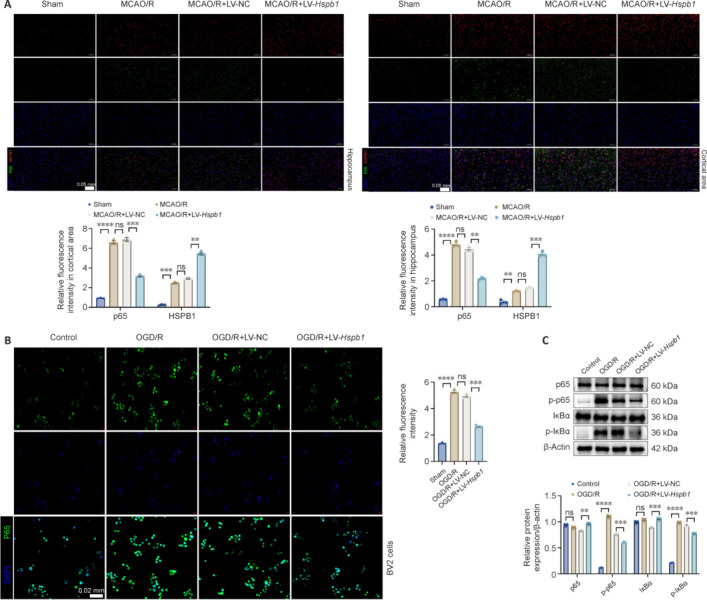
Effects of *Hspb1* overexpression on the nuclear factor (NF)-κB signaling pathway. (A) Immunofluorescence of heat shock protein beta-1 (HSPB1) and NF-κB p65 levels in brain tissue. *n* = 3 per group. (B) Immunofluorescence of p65 levels in BV-2 cells. *n* = 3 per group. (C) Western blot measurement and quantitative analysis of p65, phosphorylated (p)-p65, NF-κB inhibitor α (IκBα) and p-IκBα protein levels in BV-2 cells. *n* = 3 per group. Data are shown as mean ± SEM. **P < 0.01, ***P < 0.001, ****P < 0.0001 (one-way analysis of variance with Tukey’s *post hoc* test). Hspb1: Heat shock protein beta-1; IκBα: nuclear factor-κB inhibitory protein α; LV-Hspb1: lentiviral vectors overexpressing Hspb1; LV-NC: lentiviral vectors carrying a negative control; MCAO/R: middle cerebral artery occlusion/reperfusion; ns: not significant; OGD/R: oxygen-glucose deprivation/reoxygenation; p-: phosphorylated.

Previous studies have reported a strong correlation between the NF-κB pathway and ferroptosis (Lee and Hyun, 2023; Zhang et al., 2023). In the present study, *Hspb1* overexpression significantly downregulated p65 protein expression in both the cortical and striatal regions, thereby providing deeper insights into the underlying molecular mechanisms (**Figure 6A**). This observation suggests that *Hspb1* may function as a negative modulator of the NF-κB pathway during cerebral I/R injury. Consistent with our *in vivo* observations, *Hspb1* overexpression also suppressed p65 expression in BV-2 cells (**Figure 6B**). Moreover, increased levels of p-p65 and p65 proteins were observed after OGD/R treatment (**Figure 6C**), indicating the activation of this pro-inflammatory signaling pathway under conditions mimicking cerebral I/R injury. Importantly, when LV-*Hspb1* was transfected into BV-2 cells subjected to OGD/R insult, this pathway was effectively inhibited via the suppression of both p65 (*P* < 0.001) and p-p65 (*P* < 0.001) protein expression. Together, these findings suggest that *Hspb1* may play a crucial role in regulating cell ferroptosis through inhibition of the NF-κB pathway during cerebral I/R injury.

### A nuclear factor-κB agonist reverses the protective effects of heat shock protein beta-1 and nullifies its regulatory role in ferroptosis *in vitro*

**Figure 7:**
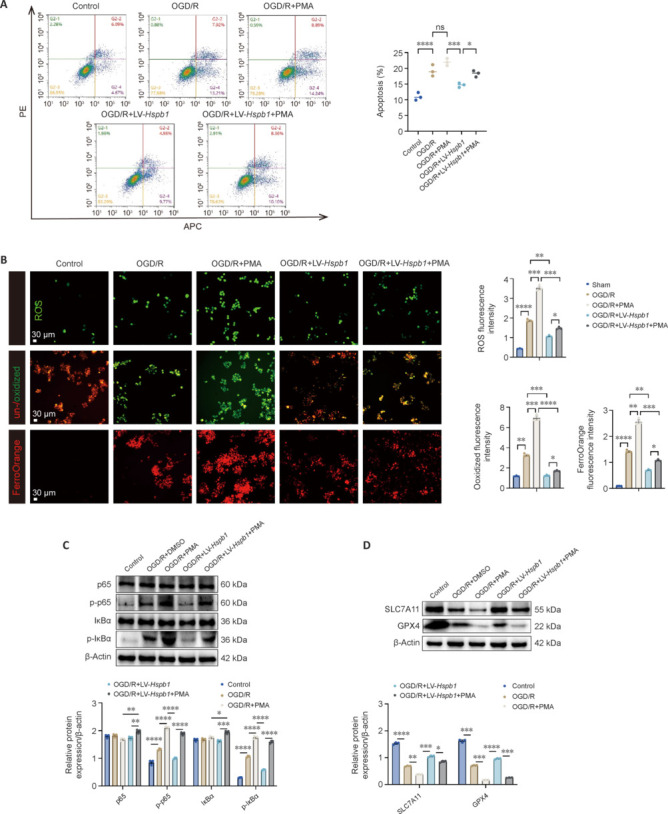
*In vitro* regulation of the nuclear factor (NF)-κB agonist phorbol 12-myristate 13-acetate (PMA) on Hspb1 protection, ferroptosis-associated protein expression, and activation of the NF-κB pathway. (A) Representative figures and quantitative analysis of apoptosis, measured using annexin-V/propidium iodide (PI) staining combined with flow cytometry in BV-2 cells. *n* = 3 per group. (B) Representative fluorescence images of intracellular reactive oxygen species (ROS) activity (row 1), C11-BODIPY (row 2), and intracellular Fe^2+^ levels (row 3) in BV-2 cells. *n* = 3 per group. (C) Western blot analysis was used to measure and quantitatively analyze the protein levels of NF-κB p65, p-p65, NF-κB inhibitor α (IκBα), and p-IκBα in BV-2 cells. *n* = 3 per group. (D) Western blot analysis was used to measure and quantitatively analyze the levels of cystine/glutamate transporter SLC7A11 and glutathione peroxidase (GPX4) proteins in BV-2 cells. n = 3 per group. Data are shown as mean ± SEM. *P < 0.05, **P < 0.01, ***P < 0.001, ****P < 0.0001 (one-way analysis of variance with Tukey’s *post hoc* test). GPX4: Glutathione peroxidase 4; Hspb1: heat shock protein beta-1; IκBα: nuclear factor-κB inhibitory protein α; LV-Hspb1: lentiviral vectors overexpressing Hspb1; LV-NC: lentiviral vectors carrying a negative control; OGD/R: oxygen-glucose deprivation/reoxygenation; PMA: phorbol 12-myristate 13-acetate (NF-κB agonist); ROS: reactive oxygen species.

The protective effects of *Hspb1* were abrogated by the NF-κB agonist PMA (80 nM, 24 hours), as evidenced by a lower apoptotic rate (*P* = 0.05) and higher oxidative stress levels (**Figure 7A** and **B**). Additionally, *Hspb1* overexpression in the LV-*Hspb1* group had an inhibitory effect on the NF-κB signaling pathway following MCAO/R. This led to decreased levels of p-p65 and p-NF-κB (**Figure 7C**). Importantly, treatment with PMA not only reversed the protective effects of *Hspb1* but also significantly elevated the protein expression levels of p-p65 (*P* < 0.0001) and p-NF-κB (*P* < 0.0001) in OGD/R cells. Additionally, PMA downregulated the ferroptosis marker GPX4 (*P* < 0.0001) in OGD/R cells (**Figure 7D**). Collectively, these findings suggest that the protective effects of *Hspb1* against ferroptosis are dependent on activation of the NF-κB pathway, and indicate that stimulation with an NF-κB agonist can reverse these *Hspb1*-induced protective effects.

## Discussion

The present study provides novel evidence that *Hspb1* exerts dual regulatory effects on ferroptosis and microglial polarization in the context of I/R injury. By integrating *in vivo* and *in vitro* models, we have demonstrated that *Hspb1* overexpression not only inhibits key molecular markers of ferroptosis (including Fe^2+^ accumulation, ROS, and MDA) but also promotes a phenotypic shift in microglia toward an anti-inflammatory state. These findings advance the current understanding of the neuroprotective role of *Hspb1* beyond what has been previously reported. Our study diverges mechanistically, highlighting a shift in mouse expression and apoptotic signaling. These differences likely reflect the tissue-specific and temporal variability of *Hspb1*-mediated responses under ischemic conditions, suggesting a relatively broad regulatory repertoire.

Ferroptosis has been linked to the development and progression of various diseases, such as cancer and I/R injury. In addition to ferroptosis, microglia play a crucial role in the progression of neuroinflammation during I/R injury. Although IBA1 reflects microglial activation, microglia exhibit diverse functional profiles based on the injury context. CD86 is associated with pro-inflammatory microglia, and contributes to neuroinflammation through cytokines such as TNF-α and IL-6. By contrast, CD206 is associated with anti-inflammatory microglia, which promote tissue repair and neuroprotection. Microglial phenotypes are dynamic and change with injury severity, duration, and location. They may initially adopt a pro-inflammatory role to limit damage, and later transition to an anti-inflammatory state for repair (Guo et al., 2025). In the present study, LV-*Hspb1* treatment increased CD206^+^ microglia, suggesting that *Hspb1* may promote neuroprotection by shifting microglia toward an anti-inflammatory state. Modulating microglial activation from a pro-inflammatory state to an anti-inflammatory state has therapeutic potential because chronic neuroinflammation worsens secondary damage after stroke. Thus, strategies that promote inflammation resolution and repair are crucial for recovery. Together, our findings suggest that *Hspb1* overexpression may serve as a therapeutic approach for mitigating cerebral damage through this mechanism. Additionally, our findings on the protective role of *Hspb1* in ischemic stroke through the inhibition of ferroptosis and the activation of microglia align with the results of Dai and Hu (2022), which also demonstrated the neuroprotective effects of *Hspb1* in hypoxic/ischemic brain injury. Both studies highlighted ferroptosis as a key target, although Dai and Hu emphasized the regulation of glucose-6-phosphate dehydrogenase by *Hspb1*. This difference in focus suggests that *Hspb1* may modulate multiple pathways to confer its protective effects in ischemic conditions, thus warranting further investigations into the broader mechanisms at play.

In the context of cerebral I/R, ferroptosis is a complex process that is characterized by various molecular and cellular events. One prominent feature of ferroptosis in cerebral I/R involves the accumulation of iron (Fe^2+^), which leads to iron overload within brain tissue. This excess iron can promote the generation of ROS and MDA, both of which are markers of oxidative stress (Zhao et al., 2023). Furthermore, during cerebral I/R, GPX4 and SLC7A11 expression levels are upregulated (Mao et al., 2022; Sanguigno et al., 2023). GPX4 plays a crucial role in protecting cells against lipid peroxidation by reducing lipid hydroperoxides to their corresponding alcohols. By contrast, SLC7A11 acts as a cystine-glutamate antiporter that regulates intracellular GSH levels (Rochette et al., 2022). GSH serves as an important antioxidant defense system within cells. However, despite these compensatory mechanisms, our study revealed that MCAO/R mice exhibited decreased levels of GPX4, SLC7A11, SOD, and GSH. This finding suggests that during cerebral I/R injury, there is a disruption in the delicate balance between pro-oxidant and antioxidant systems. The observed increase in Fe^2+^, ROS, and MDA content in brain tissue further supports the occurrence of ferroptosis during cerebral I/R. Ferroptosis has been implicated as a potential mechanism of neuronal damage following stroke or revascularization procedures. Understanding the underlying molecular pathways involved in ferroptosis may thus provide valuable insights for developing therapeutic strategies aimed at mitigating cerebral I/R-induced brain injury.

During ischemic injury, the antioxidant response prompts an increase in HSPs and chaperones to suppress signaling pathways that promote programmed cell death (Chelluboina et al., 2014). HSPB1 (also known as HSP27) is a member of the small HSP family and displays high responsiveness under stressful conditions (Liu et al., 2019); its adaptability during ischemia is particularly noteworthy. The expression pattern of *Hspb1* in ischemia varies among different types of cells, distributions, and timeframes, thereby playing a protective role in mitigating damage caused by MCAO-induced cerebral ischemia. In the present study, transduction with LV-*Hspb1* demonstrated that *Hspb1* overexpression effectively alleviated cerebral I/R-induced injury and inhibited ferroptosis in MCAO/R mice. These findings highlight the important involvement of microglial polarization and ferroptosis in cerebral I/R and align with existing literature on this subject. Moreover, our findings indicate that *Hspb1* influences the complex behavior of microglia following ischemic injury. By highlighting the reduction of pro-inflammatory activation, we have provided insights into the dynamic roles that microglia play in neuroprotection and repair. This further underscores the importance of understanding microglial plasticity for the development of effective therapeutic strategies.

Microglia-derived chemokines attract immune cells from the bloodstream into brain tissue, thus facilitating communication with resident immune cells and contributing to an excessive production of inflammatory cytokines (Greenhalgh et al., 2020; Guo et al., 2023). TNF-α, iNOS, IL-1β, and IL-6 are among the classical inflammatory mediators that have been extensively studied. Following ischemic stroke, microglia primarily release TNF-α, which reaches its peak levels at 12–24 hours after stroke. This cytokine binds to its receptors and directly triggers neuronal cell death (Meng et al., 2019). HSPB1 plays an important role in promoting drug resistance by inhibiting chemotherapy-induced ferroptosis in breast cancer through activation of the NF-κB signaling pathway (Liang et al., 2023). The NF-κB transcription factor regulates the expression of various cytokines. In the current study, we investigated the effects of *Hspb1* overexpression on inflammatory cytokines and apoptosis-related proteins. The LV-*Hspb1* group exhibited lower TNF, IL-6, and IL-1β levels compared with the control group. This finding suggests that *Hspb1* overexpression inhibits the expression of these pro-inflammatory cytokines. Although reducing inflammatory cytokine levels is often regarded as beneficial, many cytokines exhibit biphasic or even polyphasic roles in ischemic stroke. That is, their effects depend on the timing, spatial context, and severity of the injury. For example, IL-6 deletion in MCAO model mice does not confer protection against ischemic damage (Famakin, 2014). IL-6 has also been reported to act as a neurotrophic factor, improving functional outcomes and reducing infarct size. Similarly, TNF-α, which is commonly considered a pro-inflammatory cytokine, also has neuroprotective effects by reducing the production of nitric oxide and free radicals (Figiel, 2008). Together, these findings underscore the complexity of the immune response to stroke injury. Although excessive and prolonged inflammation can exacerbate tissue damage, certain cytokines may promote repair and recovery during later stages. Thus, the modulation of immune responses, rather than their complete suppression, may represent the most effective therapeutic approach.

Through additional experiments using PMA as an agonist for NF-κB signaling pathway activation, we observed that treatment with PMA reversed the suppressive effect of *Hspb1* on these cytokines. This result indicates that NF-κB may play a role in mediating the inhibitory action of *Hspb1* on inflammatory responses. Moreover, our results indicate that *Hspb1* overexpression also downregulates apoptosis-related proteins, including BAX, CASP3, and CASP9. These proteins are key regulators in apoptotic pathways. Additionally, components of the NF-κB signaling pathway, such as p65 and NF-ΚB, were also noted to be downregulated upon *Hspb1* overexpression. Overall, our study provides evidence for the anti-inflammatory properties of *Hspb1* through its ability to inhibit pro-inflammatory cytokine production and regulate apoptotic protein expression via the modulation of NF-κB signaling pathway activity.

Our study has several inherent limitations. First, the use of exclusively male mice might restrict the generalizability of our findings to human clinical conditions. Although there are sex differences in various physiological and pathological processes, including neurodevelopment and neurodegenerative diseases, our study focused solely on male mice because of practical considerations such as cost and time constraints. However, future research endeavors should aim to include both male and female animals to provide a more comprehensive understanding of the underlying mechanisms. Second, the use of an immortalized cell line rather than primary cortical neurons might compromise the representation of true environmental conditions and tissue states within animal models. Although immortalized cell lines offer advantages such as ease of maintenance and reproducibility, they may not fully capture the complexity and heterogeneity that are observed in primary cells. Furthermore, previous studies have reported that *Hspb1* expression is upregulated in ischemic stroke, suggesting its potential role in neuroprotection. However, our findings contrast with these previous results. This discrepancy may be caused by differences in experimental models, ischemic duration, or other contextual variables that affect *Hspb1* expression dynamics. Further research is therefore needed to elucidate the specific regulatory mechanisms of *Hspb1* in ischemic stroke—particularly, to understand how contextual factors can influence its expression and protective functions.

In conclusion, *Hspb1* inhibits ferroptosis and microglial inflammation in stroke by reducing oxidative stress and modulating p65 signaling. It also protects neural cells, promotes tissue repair, and offers potential therapeutic applications. Overexpressing *Hspb1* maintains cell viability and suppresses neuroinflammation via GPX4/NF-κB pathway repression, thus presenting a promising stroke treatment strategy.

## Additional files:

***Additional Figure 1:*** *Laser speckle contrast imaging reveals cerebral blood flow changes during ischemia/reperfusion injury.*

Additional Figure 1 Laser speckle contrast imaging reveals cerebral blood flow changes during ischemia/reperfusion injury.
Representative pseudocolor laser speckle contrast imaging showing the cortical surface of a mouse brain during the reperfusion phase following transient middle cerebral artery occlusion. Warm colors (red/yellow) indicate regions of higher perfusion, whereas cooler colors (blue) denote hypoperfused areas. The image highlights distinct vascular architecture, with reduced perfusion in the ischemic core and the partial restoration of blood flow in the penumbra region.

***Additional Figure 2:*** *Hspb1 lentivirus sequence.*

Additional Figure 2 *Hspb1* lentivirus sequence.
Plasmid maps of the lentiviral vectors that were used to study gene overexpression. The structural compositions and functional elements of the two different expression plasmids—LV-CMV-mCS-3flag-EF1-puro (upper image, length: 8191 bp) and LV-CMV-mhspb1-3flag-EF1-puro (lower image, length: 8790 bp)—can be observed.

***Additional Figure 3:*** *Expression of Hspb1 in MCAO/R mice detected by quantitative reverse transcription-polymerase chain reaction.*

Additional Figure 3 Expression of *Hspb1* in MCAO/R mice detected by quantitative reverse transcription-polymerase chain reaction.
β-actin was used as the endogenous control. *n* = 3. ^***^P < 0.001. Hspb1: Heat shock protein beta-1; LV-Hspb1: lentiviral vectors overexpressing Hspb1; LV-NC: lentiviral vectors carrying a negative control; MCAO/R: middle cerebral artery occlusion/reperfusion.

***Additional Figure 4:*** *Expression of HSPB1 in MCAO/R mice detected by western blotting.*

Additional Figure 4 Expression of HSPB1 in MCAO/R mice detected by western blotting.
^***^P < 0.001. HSPB1: Heat shock protein beta-1; LV-Hspb1: lentiviral vectors overexpressing Hspb1; LV-NC: lentiviral vectors carrying a negative control; MCAO/R: middle cerebral artery occlusion/reperfusion.

***Additional Figure 5:*** *Co-localization of HSPB1 and IBA1 in the peri-infarct cortex following MCAO/R injury.*

Additional Figure 5 Co-localization of HSPB1 and IBA1 in the peri-infarct cortex following MCAO/R injury.
Representative immunofluorescence images showing the co-localization of HSPB1 (red) with the microglial marker IBA1 (green) in the peri-infarct region of the mouse brain after MCAO/R. Nuclear staining was performed with 4',6-diamidino-2-phenylindole (DAPI; blue). Merged images reveal cells co-expressing IBA1 and HSPB1 (yellow, arrows), indicating HSPB1 upregulation in activated microglia after ischemic injury. Arrows represent positive cells. DAPI: 4',6-Diamidino-2-phenylindole; HSPB1: heat shock protein beta-1; IBA-1: ionized calcium-binding adapter molecule 1; MCAO/R: middle cerebral artery occlusion/reperfusion.

***Additional Table 1:*** *Sequences of Hspb1 lentivirus.*

***Additional Table 2:*** *Primer sequences for quantitative reverse transcription-polymerase chain reaction.*

## Data Availability

*All relevant data are within the paper and its Additional files*.
