# Supplementary figures and images for: Hspb1 inhibits microglial ferroptosis and pro-inflammatory activation to alleviate cerebral ischemia/reperfusion injury in mice
Source: Neural Regen Res. 2025 Aug 13;21(7):3225–37. doi: 10.4103/NRR.NRR-D-24-01532 (PMC13384247; doi:10.4103/NRR.NRR-D-24-01532)

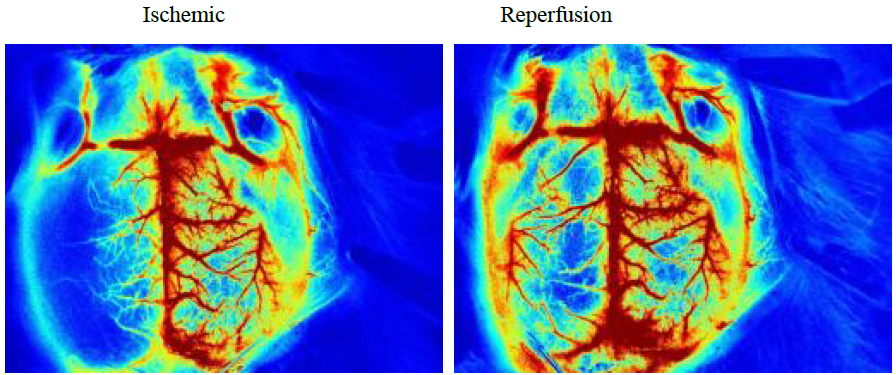

Supplement: Supplementary file 1 [file NRR-21-3225_Suppl1.tif]

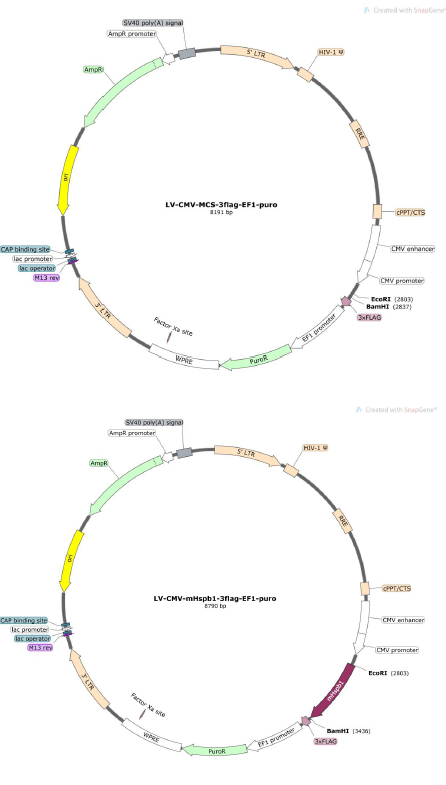

Supplement: Supplementary file 2 [file NRR-21-3225_Suppl2.tif]

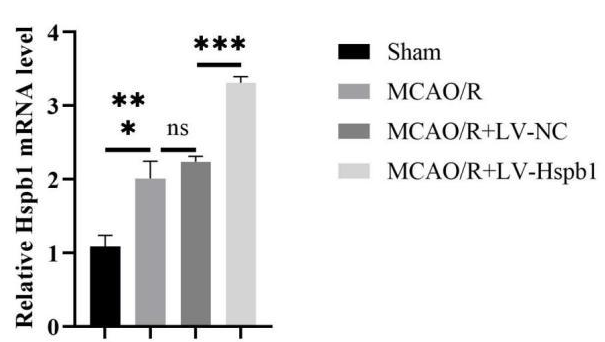

Supplement: Supplementary file 3 [file NRR-21-3225_Suppl3.tif]

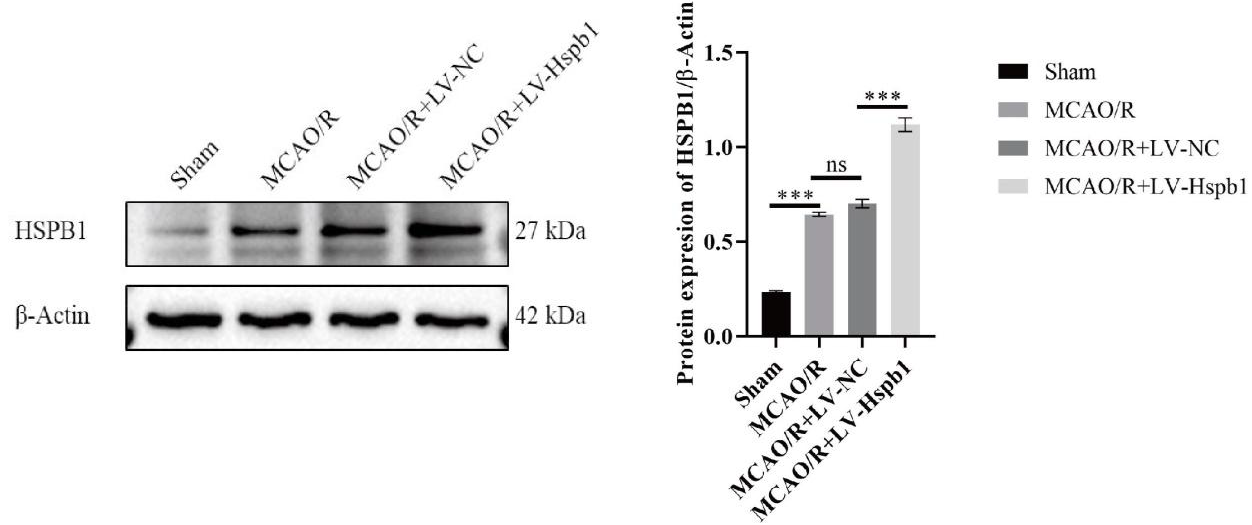

Supplement: Supplementary file 4 [file NRR-21-3225_Suppl4.tif]

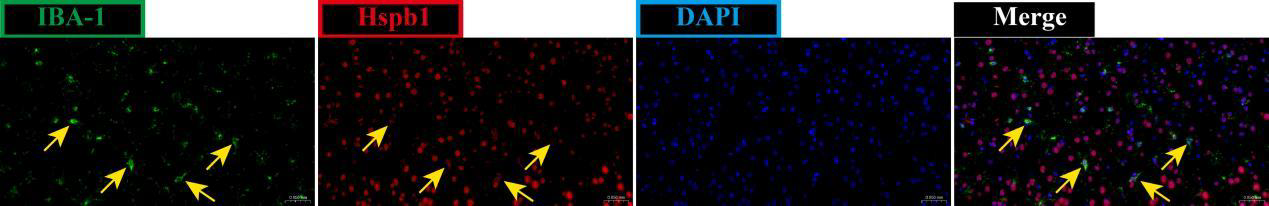

Supplement: Supplementary file 5 [file NRR-21-3225_Suppl5.tif]
